# Association of serum water-soluble vitamin exposures with the risk of metabolic syndrome: results from NHANES 2003-2006

**DOI:** 10.3389/fendo.2023.1167317

**Published:** 2023-05-12

**Authors:** Xun Pei, Junjie Yao, Simiao Ran, Haifei Lu, Shuo Yang, Yini Zhang, Miyuan Wang, Heyuan Shi, Aihua Tan

**Affiliations:** ^1^ Hubei Provincial Hospital of TCM (Affiliated Hospital of Hubei University of Chinese Medicine), Wuhan, Hubei, China; ^2^ College of Acupuncture and Tuina, Changchun University of Chinese Medicine, Changchun, Jilin, China; ^3^ Department of Gastroenterology, HuangGang Hospital of Traditional Chinese Medicine (TCM), Affiliated to Hubei University of Chinese Medicine, Huanggang, Hubei, China; ^4^ Basic Medicine College, Hubei University of Chinese Medicine, Wuhan, Hubei, China; ^5^ School of Management Beijing, University of Chinese Medicine, Beijing, China; ^6^ Dongzhimen Hospital of Beijing University of Chinese Medicine / Postdoctoral Station of Beijing University of Chinese Medicine, Beijing, China

**Keywords:** vitamin, metabolic syndrome, weighted quantile sum regression, NHANES, co-exposure

## Abstract

**Introduction:**

Existing evidence suggests an association between certain vitamins and metabolic syndrome (MetS), but few epidemiological studies have focused on the effects of multivitamin co-exposure on MetS. This study aims to investigate the associations of the individual or multiple water-soluble vitamins (i.e., vitamin C (VC), vitamin B9 (VB9), and vitamin B12 (VB12)) with co-exposure to MetS, as well as the dose-response relationships among them.

**Methods:**

A cross-sectional study was conducted by employing the National Health and Examination Surveys (NHANESs) 2003-2006. Multivariate-adjusted logistic regression models were used to explore the association between individual serum water-soluble vitamins and the risk of MetS and its components, including waist circumference, triglyceride, high-density lipoprotein, blood pressure, and fasting plasma glucose. Restricted cubic splines were performed to explore the dose-response relationships among them. The quantile g-computation method was adopted to explore the associations of multiple water-soluble vitamins co-exposure with MetS risk and MetS components.

**Results:**

A total of 8983 subjects were involved in the study, of whom 1443 were diagnosed with MetS. The MetS groups had a higher proportion of participants with age ≥60 years, BMI ≥30 kg/m^2^, and insufficient physical activity. Compared with the lowest quartile, the third (OR=0.67, 95% CI: 0.48, 0.94) and highest quartiles (OR=0.52, 95%CI: 0.35, 0.76) of VC were associated with lower MetS risk. Restricted cubic splines showed negative dose-response relationships among VC, VB9 and VB12, and MetS. Regarding MetS components, higher VC quartiles were associated with lower waist circumference, triglyceride, blood pressure, and fasting plasma glucose, while higher VC and VB9 quartiles were associated with higher high-density lipoprotein (HDL). Co-exposure to VC, VB9, and VB12 was significantly inversely associated with MetS, with ORs (95% CI) of 0.81 (0.74, 0.89) and 0.84 (0.78, 0.90) in the conditional and marginal structural models, respectively. Furthermore, we found that VC, VB9, and VB12 co-exposure were negatively associated with waist circumference and blood pressure, while VC, VB9, and VB12 co-exposure were positively associated with HDL.

**Conclusion:**

This study revealed negative associations of VC, VB9, and VB12 with MetS, while the high water-soluble vitamin co-exposure was associated with a lower MetS risk.

## 1 Introduction

Metabolic syndrome (MetS) is a major health problem faced by sub-healthy populations. With the development of the global economy and the general improvement of living standards, more than one billion people are affected by MetS, becoming a global public health challenge (1). The definition of MetS varies slightly among healthcare organizations, but it can be summarized as a pathology characterized by obesity, insulin resistance, a pro-inflammatory state, hypertension, and hyperlipidemia. It may also be a high-risk metabolic factor that promotes the development of type 2 diabetes and cardiovascular disease (2, 3). Studies have shown that the pathogenesis of MetS involves a variety of factors, among which oxidative stress and inflammatory responses play major roles. In a state of nutrient overload, the normal metabolic balance is disrupted, and the excessive production of active substances causes changes in the regulation of redox metabolism, forming a vicious circle (4, 5). Most patients with MetS have subclinical symptoms that may not be well controlled with medication but can be addressed with dietary changes such as increasing fiber, vitamin-rich grains, vegetables, and fruits while reducing refined, high-sugar, and high-fat foods intake (6).

Vitamins are divided into water-soluble and fat-soluble according to their solubility. Both types are essential trace compounds involved in complex biochemical reactions and metabolic regulation of the human body. B vitamins and vitamin C (VC), as water-soluble vitamins, are generally considered to be cofactors in preventing metabolic disorders and in the functioning of various enzymes (7, 8). Recent studies have indicated the importance of water-soluble vitamins in regulating metabolism and maintaining physiological homeostasis. VC is a widely used free radical scavenging reductant that cannot be synthesized by the human body, but in the case of a normal diet, dietary intake can provide the daily required VC level of the human body (9). Thomas-Valdés S et al. demonstrated that the body of obese individuals produces low-grade inflammation and enhanced oxidative stress due to excessive nutrient intake, a state that inhibits VC absorption and promotes excessive consumption of VC (10). Abdominal obesity-induced adipocyte increase and dyslipidemia promote systemic oxidative stress, forming a vicious cycle of obesity, inflammation, and oxidative stress (11). B9 (folic acid) and B12 are important B vitamins that play critical roles in homocysteine metabolism and neurodevelopment. These two vitamins are often studied together because of their similar functions (12). Several recent studies have shown that B9 and B12 have equally profound effects on different pathological features of MetS, particularly affecting lipid metabolism and high risk of cardiovascular disease (13–16). A study based on 341 healthy Saudi women showed that low serum vitamin B12 levels were independently associated with dyslipidemia (17). This imbalance during pregnancy predisposes women to diabetes and also predisposes their children to metabolic diseases such as insulin resistance and obesity (18, 19).

Past epidemiological studies indicated the relationship between vitamin D and other fat-soluble vitamins and MetS (20). However, relatively few studies have been conducted on water-soluble vitamins. In addition, the effects of individual vitamins have often been studied and few studies have focused on the effects of multivitamin co-exposure on MetS. An earlier observational study demonstrated an association between individual concentrations of water-soluble vitamins and MetS. However, controlled trials only examined results for B1, B3, B6, and VC with small sample sizes, but did not evaluate their co-exposure effects (21). The intake of fresh vegetables and fruits has been shown to be significantly and inversely associated with the prevalence of MetS. Because these foods are rich in vitamins and phytochemicals, they can reduce oxidative stress, regulate endothelial function, and reduce insulin resistance (22). However, there is currently insufficient evidence to prove that the use of multivitamins has any effect on reducing the prevalence of metabolic syndrome.

Therefore, in this study, the relationship between circulating levels of three common water-soluble vitamins (VC, VB9, and VB12) and MetS was analyzed by using data from U.S. adults who participated in the 2003−2006 National Health and Nutrition Examination Survey (NHANES) to investigate the specific effects of individual or multi-exposure of water-soluble vitamins in serum on MetS.

## 2 Materials and methods

### 2.1 Study design and participants

This study conducted a cross-sectional study utilizing data from the NHANES 2003-2004 and 2005-2006. The NHANES was conducted using a complex, multistage probability sampling design, aiming to assess the health and nutritional status of the noninstitutionalized household population. The National Center for Health Statistics Research Ethics Review Board approved the protocol of the NHANES. Each participant signed the consent form.

**Figure 1:**
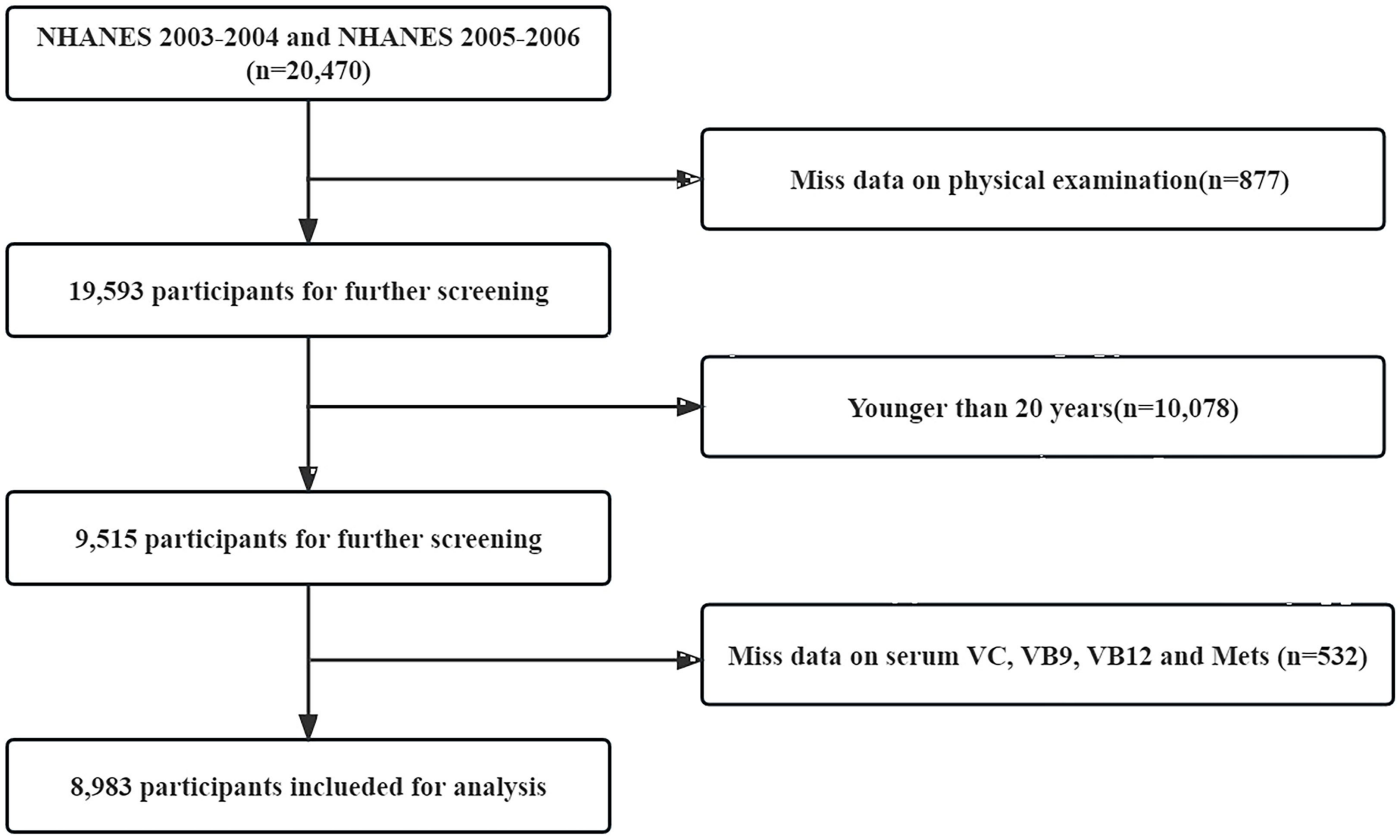
Flow chart for the selection of participants in the cross-sectional study.

All respondents that participated in the NHANES were eligible for inclusion (n=20 470). We excluded participants who only received an interview without physical examination (n=877), whose age <20 years (n=10 078), and who missed data on serum Vitamin C (VC), Vitamin B9 (VB9), Vitamin B12 (VB12), and MetS (n=532). Finally, a total of 8 983 U.S. adults were included in the present study (See **Figure 1**).

### 2.2 Exposure measurement

Exposure in this study included 3 kinds of water-soluble vitamins, namely VC, VB9 (folic acid), and VB12. Serum concentrations of VC were measured by using high-performance liquid chromatography (HPLC) and quantified by spectrophotometry. Serum concentrations of VB9 and VB12 were measured by using the radioassay kit from Bio-Rad Laboratories. Details could be found on the NHANES website (https://www.cdc.gov/nchs/nhanes/).

### 2.3 Outcome measurement

The main outcome of this study was MetS. According to the ATP III guidelines (23), clinical identification of the Mets was diagnosed when any 3 of the following occurred: (1) male waist circumference >102 cm or female waist circumference >88cm; (2) triglyceride ≥150 mg/dL; (3) high-density lipoprotein <40 mg/dL for men or <50 mg/dL for women; (4) blood pressure ≥130/≥85 mmHg; and (5) fasting glucose ≥110 mg/dL. Each component of MetS was categorized into two groups according to ATP III guidelines and was used as the secondary outcome. Waist circumference was categorized into low (<102 cm in men or <88 cm in women) and high (≥102 cm in men or ≥88 cm in women) groups; triglyceride was categorized into low (<150 mg/dL) and high (≥150 mg/dL) groups; high-density lipoprotein was categorized into low (<40 mg/dL in men or <50 mg/dL in women) and high (≥40 mg/dL in men or ≥50 mg/dL in women) groups; blood pressure was categorized into low (systolic blood pressure <130 mmHg and diastolic blood pressure<85 mmHg) and high (systolic blood pressure ≥130 mmHg or diastolic blood pressure ≥85 mmHg) groups; fasting plasma glucose was categorized into low (<110 mg/dL) and high (≥110 mg/dL) groups.

### 2.4 Covariates

Covariates included demographics and lifestyle factors. Demographic information was collected through means such as questionnaires or physical examinations, including age (20-39, 40-59, and ≥ 60 years), ethnicity (Mexican American, Hispanic, non-Hispanic White, non-Hispanic Black, and others), educational background (below high school, high school or equivalent and above high school), marital status (married/living with a partner, widowed/separated/devoiced, never married), the ratio of family income to poverty (Family PIR) and body mass index (BMI, <18.5 kg/m^2^, 18.5-24.9 kg/m^2^, 25-29.9 kg/m^2^, ≥30 kg/m^2^). Lifestyle information was collected by questionnaires or dietary interviews, including leisure-time physical activity (sedentary, insufficient, moderate, and high), drinking status (never, ever, and current), smoking status (never, ever, and current), and dietary total energy.

BMI was calculated by using weight (kg) divided by the square of height (m^2^). Leisure-time physical activity was categorized into 4 groups based on metabolic equivalent (MET)-minutes per week: sedentary (MET=0), insufficient (0<MET ≤ 500), moderate (500<MET ≤ 1000), and high (MET>1000).

### 2.5 Statistical analysis

Categorical variables were presented as numbers (percentage), and a chi-square test was adopted to compare differences between the two groups. Continuous variables that did not satisfy the normal distribution were presented as medians (interquartile ranges), and the Wilcoxon rank-sum test was used to compare differences between the two groups. Given the complex sampling design of NHANES, we employed appropriate sampling weights in our analysis.

The multivariate-adjusted logistic regression model was used to explore the associations of individual serum water-soluble vitamins with MetS risk and each component. Serum water-soluble vitamins were categorized into four groups based on corresponding quartiles and the first quartile was used as a reference. We estimated the odds ratio and corresponding 95% confidence interval in the two models. Model 1 was adjusted for general characteristics, including age, sex, ethnicity, educational background, marital status, BMI, and family PIR. Model 2 was adjusted for lifestyle factors in addition to those in model 1, including leisure time physical activity, drinking status, smoking status, and dietary total energy. Also, continuous serum water-soluble vitamins were used as exposure to performing the same analysis to test the robustness of the results.

To explore the dose-response relationships between serum water-soluble vitamins and MetS, we further performed a restricted cubic spline with three knots located at the 5^th^, 50^th,^ and 95^th^ percentiles of the vitamin concentration. Covariates adjusted were consistent with the multivariate-adjusted logistic regression model 2.

To explore the associations of water-soluble vitamins co-exposure with MetS risk and each component, we used the quantile g-computation method. Prior to the analysis, three kinds of water-soluble vitamins were log-transformed to ensure a normal distribution and then were standardized to eliminate the influence of units. We used the “qgcomp.noboot” function to obtain a positive or negative weight of each water-soluble vitamin in the contribution for qgcomp index and a conditional odds ratio. And “qgcomp.boot” function was used to assess the linearity of the total exposure effect and get a marginal odds ratio.

All data analyses were performed using SAS 9.4. All analyses were on two sides, and a *P* value of <0.05 was considered statistically significant.

## 3 Results

### 3.1 General characteristics

A total of 8983 participants were involved in the present study, 16.0% (n=1 443) of them were diagnosed with MetS according to the ATP III guidelines. Apart from sex and drinking status, the distributions of the other variables were all significantly different between the two groups. We found the proportion of participants aged ≥60 years, overweight (BMI ≥25 kg/m^2^) and with insufficient physical activity in the MetS group, were higher than that in the non-MetS group. Besides, concentrations of serum VC and VB12 in the MetS group were lower than those in the non-MetS group. No significant difference was found in serum VB9 between the two groups (**Table 1**).

**Table 1:** General characteristics of participants in NHANES 2003-2006.

Variables	Participants without Mets (n=7540)	Participants with Mets (n=1443)	*P*
Age^*^, years, n (%)			< 0.0001
20-39	3038 (40.29)	221 (15.32)	
40-59	2195 (29.11)	463 (32.09)	
≥60	2307 (30.60)	759 (52.60)	
Sex^*^, n (%)			0.2483
Men	3652 (48.44)	675 (46.78)	
Women	3888 (51.56)	768 (53.22)	
Ethnicity ^*^, n (%)			< 0.0001
Mexican American	1490 (19.76)	234 (22.45)	
Other Hispanic	237 (3.14)	38 (2.63)	
Non-Hispanic White	3875 (51.39)	793 (54.95)	
Non-Hispanic Black	1621 (21.50)	240 (16.63)	
Others	317 (4.20)	48 (3.33)	
Education^*^, n (%)			< 0.0001
Under high school	2052 (27.26)	503 (34.86)	
High school or equivalent	1818 (24.15)	370 (25.64)	
Above high school	3658 (48.59)	570 (39.50)	
Marital status^*^, n (%)			< 0.0001
Married/cohabiting	4675 (62.05)	888 (61.54)	
Widowed/divorced/separated	1568 (20.81)	425 (29.45)	
Never married	1291 (17.14)	130 (9.01)	
BMI^*^, kg/m2, n (%)			< 0.0001
< 18.5	137 (1.85)	0 (0)	
18.5-24.9	2482 (33.55)	100 (7.01)	
25.0-29.9	2640 (35.69)	445 (31.21)	
≥ 30.0	2138 (28.90)	881 (61.78)	
Physical activity^*^, n (%)			< 0.0001
Insufficient	1657 (36.28)	328 (43.21)	
Moderate	894 (19.58)	197 (25.96)	
High	2016 (44.14)	234 (30.83)	
Drinking status^*^, n (%)			0.1375
Never	942 (45.38)	241 (46.17)	
Ever	653 (31.45)	180 (34.48)	
Current	481 (23.17)	101 (19.35)	
Smoking status^*^, n (%)			< 0.0001
Never	3924 (52.10)	684 (47.43)	
Ever	1902 (25.25)	468 (32.45)	
Current	1706 (22.65)	290 (20.11)	
Family PIR*, median (IQR)	2.34 (1.24,4.18)	2.07 (1.17,3.89)	0.0010
Total energy intake^#^, kcal/day, median (IQR)	1972.50 (1495.00, 2581.00)	1802.50 (1374.00, 2353.50)	< 0.0001
Vitamin C^#^, μmol/L, median (IQR)	55.10 (36.30, 70.40)	47.70 (27.80, 64.70)	< 0.0001
Vitamin B9^#^, nmol/L, median (IQR)	26.30 (18.80,36.90)	26.30 (18.80, 37.60)	0.5344
Vitamin B12^#^, pmol/L, median (IQR)	345.38 (259.04, 459.04)	331.36 (245.02, 443.54)	0.0004

NHANES, the National Health and Nutrition Examination Survey; BMI, body mass index; Family PIR, ratio of family income to poverty; IQR, interquartile range; Mets, metabolic syndromes.

Physical activity was categorized into four groups based on metabolic equivalent (MET)-minutes per week: sedentary (MET=0), insufficient (0<MET<500), moderate (500≤MET<1000) and high (MET≥1000). But no participant was categorized into “sedentary” group.

*, P values were calculated using t-test; #, P values were calculated using Wilcoxon rank-sum test.

### 3.2 Association between individual water-soluble vitamins and MetS

The results of the multivariate-adjusted logistic regression model were shown in **Table 2**. After adjusting for all covariates, compared with the lowest quartile, the third (OR=0.67, 95%CI: 0.48, 0.94) and highest quartiles (OR=0.52, 95%CI: 0.35, 0.76) of VC were found to be associated with 33% and 48% reduction of MetS risk, respectively. No significant associations of VB9 and VB12 with MetS risk were found.

**Table 2:** Associations of serum water-soluble vitamins with metabolic syndromes in NHANES 2003-2006 participants.

Vitamin	Quantile 1	Quantile 2	Quantile 3	Quantile 4
Model1				
Vitamin C	1.00	0.84 (0.62, 1.15)	0.66 (0.48, 0.91)	0.52 (0.37, 0.73)
Vitamin B9	1.00	0.82 (0.59, 1.15)	0.99 (0.69, 1.42)	0.83 (0.61, 1.13)
Vitamin B12	1.00	1.08 (0.83, 1.42)	0.84 (0.65, 1.08)	0.93 (0.71, 1.23)
Model2				
Vitamin C	1.00	0.87 (0.64, 1.19)	0.67 (0.48, 0.94)	0.52 (0.35, 0.76)
Vitamin B9	1.00	0.81 (0.57, 1.15)	0.94 (0.65, 1.37)	0.82 (0.59, 1.14)
Vitamin B12	1.00	1.06 (0.79, 1.42)	0.84 (0.64, 1.10)	0.90 (0.67, 1.20)

NHANES, the National Health and Nutrition Examination Survey.

All estimates were calculated by multivariable logistic regression model, and results were expressed as odds ratio (95% confidence interval). Covariates adjusted in model 1 included age, sex, ethnicity, education, marital status, body mass index and ratio of family income to poverty. Covariates adjusted in model 2 included leisure time physical activity, dietary energy, drinking status and smoking status in addition to those in model 1.

Compared with the lowest quartile, the third quartile of VC was associated with lower waist circumference (OR=0.63, 95%CI: 0.43, 0.93), blood pressure (OR=0.63, 95%CI: 0.49, 0.80), and fasting plasma glucose (OR=0.70, 95%CI: 0.52, 0.94); the highest quartile of VC was associated with lower triglyceride (OR=0.73, 95%CI: 0.55, 0.96) and blood pressure (OR=0.60, 95%CI: 0.43, 0.83), and higher high-density lipoprotein (OR=0.47, 95%CI: 0.32, 0.71); and the highest quartile of VB9 was associated with higher high-density lipoprotein (OR=0.68, 95%CI: 0.47, 0.97) (model S1).

### 3.3 Dose-response relationship between individual water-soluble vitamins and MetS

**Figure 2:**
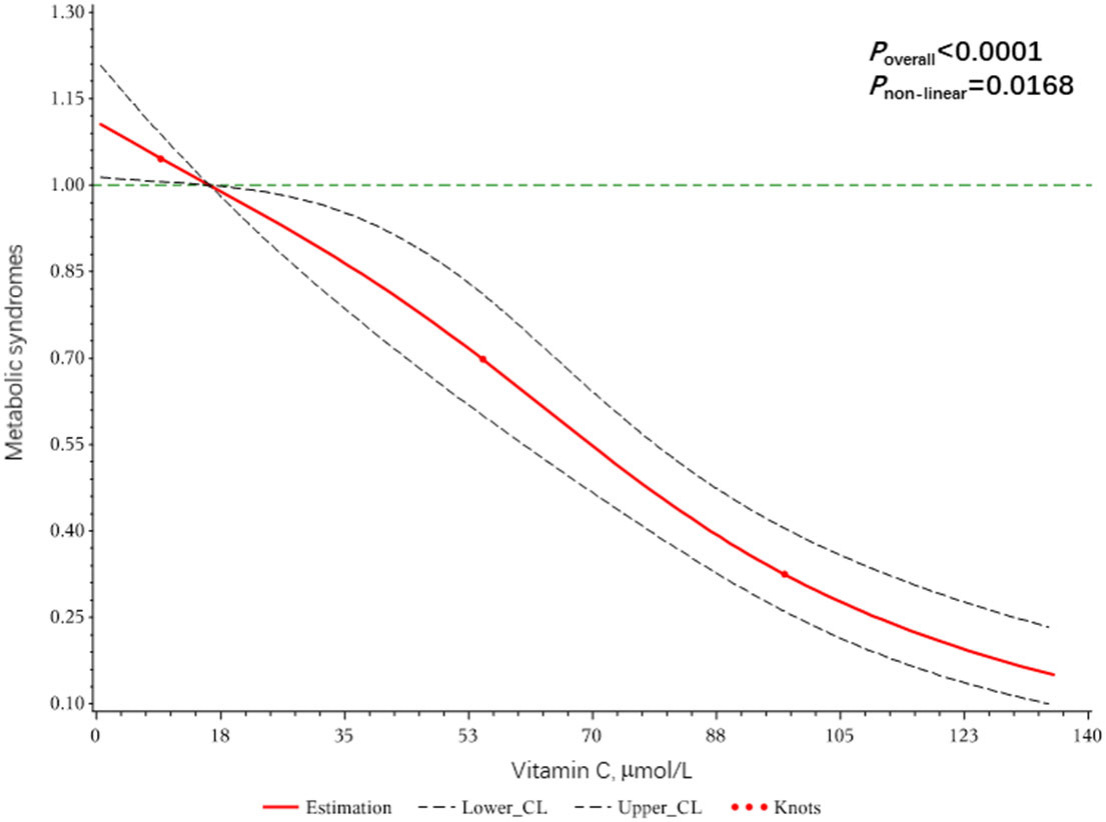
The dose-response relationship between serum Vitamin C and metabolic syndrome risk.

**Figure 3:**
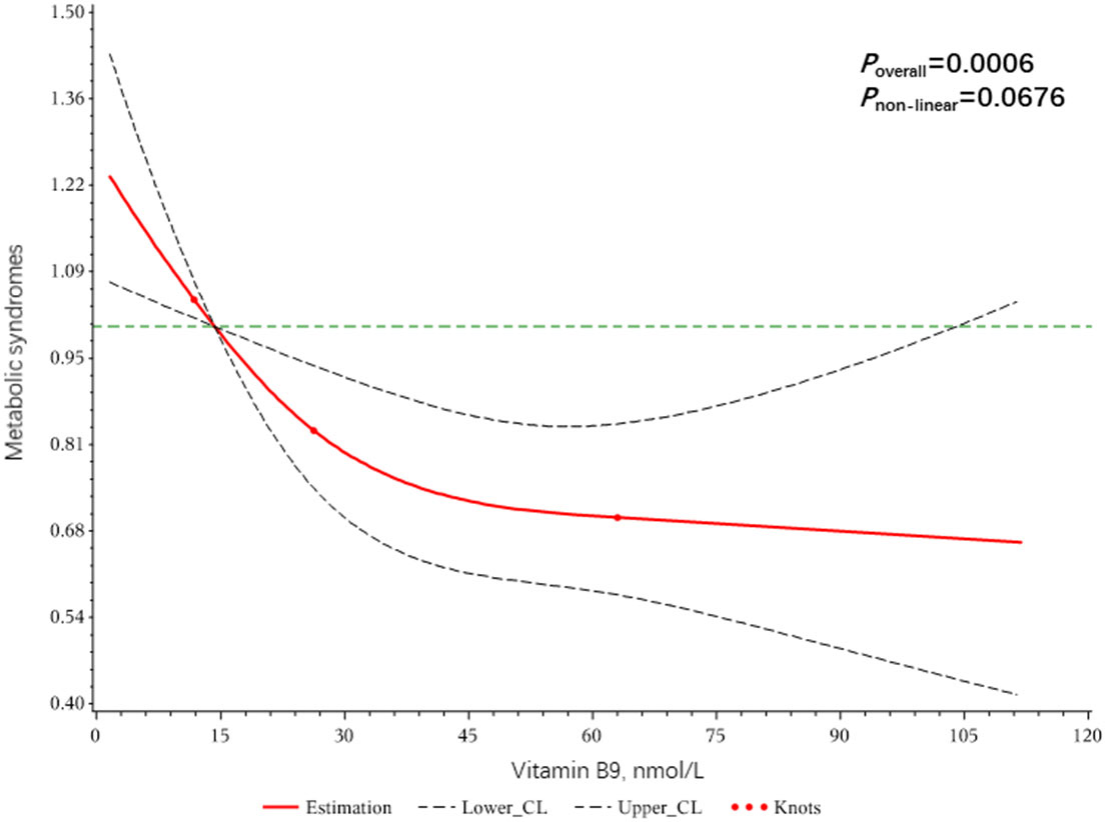
The dose-response relationship between serum Vitamin B9 and metabolic syndrome risk.

**Figure 4:**
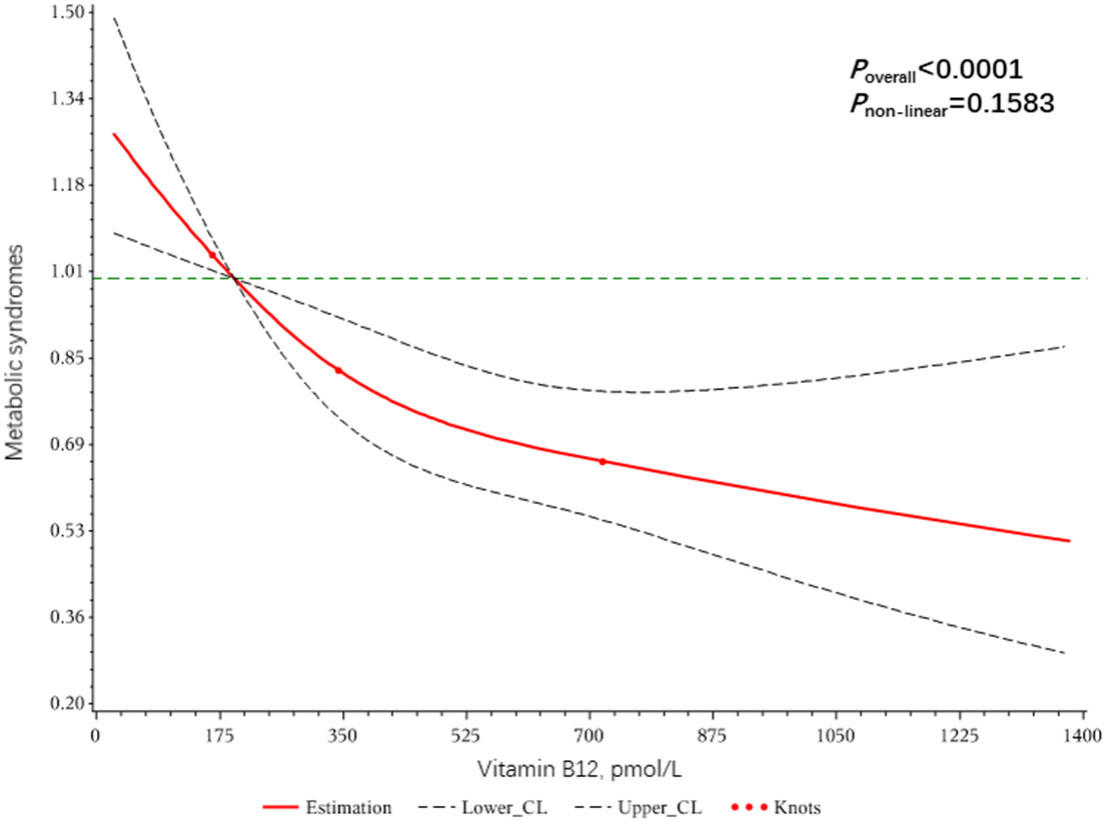
The dose-response relationship between serum Vitamin B12 and metabolic syndrome risk.

Dose-response relationships of VC, VB9, and VB12 with Mets were shown in **Figures 2**–**4**. We found a non-linear dose-response relationship between VC and MetS (**Figure 2**) and negative non-linear dose-response relationships between VB9 (**Figure 3**), VB12 (**Figure 4**), and MetS, suggesting MetS risk increased with increasing concentrations of serum VC, VB9, and VB12.

### 3.4 Association between water-soluble vitamins co-exposure and MetS

**Figure 5:**
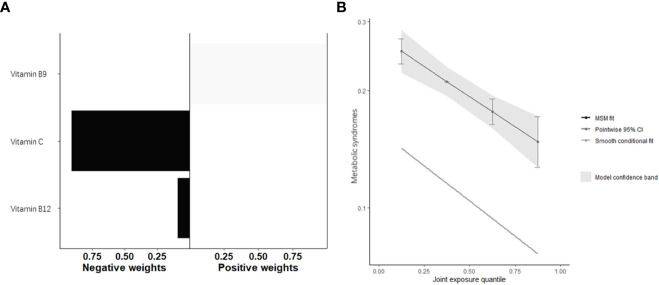
Quantile g-computation model regression index weighs **(A)** and joint effect **(B)** (95% confidence interval) of fat-soluble vitamins (i.e., Vitamin C, Vitamin B9, and Vitamin B12) on metabolic syndrome.

After adjusting for all covariates, the water-soluble vitamin as whole was significantly associated with Mets, with OR=0.81 (95%CI: 0.74, 0.89) in the conditional model and OR=0.84 (95%CI: 0.78, 0.90) in the marginal structural model for every quartile increase in fat-soluble vitamin concentrations (**Table 3**, **Figure 5B**). **Figure 5A** and **Table 4** provided the estimated weight of each water-soluble vitamin’s contribution to the qgcomp index.

**Table 3:** Association between water -soluble vitamins co-exposure and metabolic syndromes.

Models	OR (95% CI)	*P* value
Metabolism syndromes		
Conditional model	0.81 (0.74, 0.89)	< 0.0001
Marginal structural model	0.84 (0.78, 0.90)	< 0.0001
Waist Circumference		
Conditional model	0.89 (0.81, 0.98)	0.0193
Marginal structural model	0.95 (0.92, 0.99)	0.027
Triglyceride		
Conditional model	0.98 (0.89,1.08)	0.6726
Marginal structural model	0.98 (0.90, 1.07)	0.6672
High-density lipoprotein		
Conditional model	0.79 (0.74, 0.85)	<0.0001
Marginal structural model	0.81 (0.76, 0.86)	<0.0001
Blood pressure		
Conditional model	0.91 (0.85, 0.98)	0.01049
Marginal structural model	0.93 (0.87, 0.99)	0.01808
Fasting plasma glucose		
Conditional model	0.95 (0.84, 1.07)	0.3832
Marginal structural model	0.96 (0.87, 1.05)	0.3724

OR, odds ratio; CI, confidence interval.

Concentrations of serum Vitamin C, Vitamin B9 and Vitamin B12 were log-transformed and then were standardized.

Each component of metabolism syndromes was categorized based on NCEP: ATP III guidelines. Waist circumference was categorized into low (<102 cm in men or <88 cm in women) and high (≥102 cm in men or ≥88 cm in women) groups, and low group was used as reference; triglyceride was categorized into low (<150 mg/dL) and high (≥150 mg/dL) groups, and low group was used as reference; high-density lipoprotein was categorized into low (<40 mg/dL in men or <50 mg/dL in women) and high (≥40 mg/dL in men or ≥50 mg/dL in women) groups, and high group was used as reference; blood pressure was categorized into low (systolic blood pressure <130 mmHg and diastolic blood pressure<85 mmHg) and high (systolic blood pressure ≥130 mmHg or diastolic blood pressure ≥85 mmHg) groups, and low group was used as reference; fasting plasma glucose was categorized into low (<110 mg/dL) and high (≥110 mg/dL) groups, and low group was used as reference.

**Table 4:** Weights of each serum vitamin in the association of water-soluble vitamins co-exposure with metabolic syndromes.

Serum fat-soluble Vitamins	Positive weight	Negative weight
Metabolism syndromes		
Vitamin C	0	0.909
Vitamin B9	1.000	0
Vitamin B12	0	0.091
Waist Circumference		
Vitamin C	0	0.404
Vitamin B9	1.000	0
Vitamin B12	0	0.596
Triglyceride		
Vitamin C	0	0.575
Vitamin B9	1.000	0
Vitamin B12	0	0.425
High-density lipoprotein		
Vitamin C	0	0.665
Vitamin B9	0	0.335
Vitamin B12	1.000	0
Blood pressure		
Vitamin C	0	0.906
Vitamin B9	0	0.042
Vitamin B12	0	0.052
Fasting plasma glucose		
Vitamin C	1.000	0
Vitamin B9	0	0.594
Vitamin B12	0	0.406

Concentrations of serum Vitamin C, Vitamin B9 and Vitamin B12 were log-transformed and then were standardized.

Each component of metabolism syndromes was categorized based on NCEP: ATP III guidelines. Waist circumference was categorized into low (<102 cm in men or <88 cm in women) and high (≥102 cm in men or ≥88 cm in women) groups, and low group was used as reference; triglyceride was categorized into low (<150 mg/dL) and high (≥150 mg/dL) groups, and low group was used as reference; high-density lipoprotein was categorized into low (<40 mg/dL in men or <50 mg/dL in women) and high (≥40 mg/dL in men or ≥50 mg/dL in women) groups, and high group was used as reference; blood pressure was categorized into low (systolic blood pressure <130 mmHg and diastolic blood pressure<85 mmHg) and high (systolic blood pressure ≥130 mmHg or diastolic blood pressure ≥85 mmHg) groups, and low group was used as reference; fasting plasma glucose was categorized into low (<110 mg/dL) and high (≥110 mg/dL) groups, and low group was used as reference.

## 4 Discussion

The relationship between circulating levels of three water-soluble vitamins and MetS was evaluated using data from two consecutive NHANES periods. The researchers considered the specific individual and co-exposure effects of VC, B9, and B12 on MetS separately. The risk of MetS decreased with increased circulating levels of water-soluble vitamins. Also, the third and highest quartiles of VC were associated with a 33% and 48% reduced risk of MetS compared with the lowest quartile, respectively. A nonlinear dose-response relationship between VC and MetS and a linear dose-response relationship between B9, B12, and MetS were also noted. The findings clearly suggest the effects of co-exposure to water-soluble vitamins on MetS.

### 4.1 VC and MetS

Among the three water-soluble vitamins studied, a significant negative association between serum VC level and MetS risk was observed. VC, known as L-ascorbic acid, is a vitamin that relies entirely on food intake and is widely used as a common antioxidant (24). Adults with higher levels of VC in their blood generally have better metabolic health, such as body mass index, waist circumference, and blood lipids. In middle-aged and older populations, higher VC blood levels are associated with lower levels of cognitive impairment (25, 26). Previous epidemiological studies suggest that VC deficiency increases increased the risk of MetS in adults and leads to an increased incidence of type 2 diabetes (27). A meta-analysis of observational studies with 110,771 participants also concludes that both diet and circulating VC levels are negatively associated with MetS (28). These findings are consistent with our results. MetS per se has been shown to result in reduced plasma VC, and VC deficiency in turn leads to the development of MetS. Because VC deficiency will not only lead to decreased cholesterol excretion and damage hepatic lipid homeostasis, but also reduce the expression and activity of various antioxidant enzymes, increase oxidative stress markers, and thus lose the protective effect on hepatic protein and lipid oxidation (29). MetS may repeatedly produce metabolic endotoxemia due to impaired intestinal barrier function due to excess nutrition, which is a vicious cycle that decreases VC absorption while increasing inflammation and oxidative damage (30). Increasing dietary VC intake is beneficial for improving glucose metabolism and liver function, improving lipid distribution by reducing triglycerides and total cholesterol, and increasing the bioavailability of multiple antioxidant vitamins by relieving inflammation in the gut-liver axis (31–33). In addition, our study observed a nonlinear dose-response relationship between VC and MetS, although the characteristics were not significant.

### 4.2 Vitamin B9 and MetS

A linear dose-response relationship was noted between the circulating level of vitamin B9 and MetS. There are some studies indicating the prevalence of vitamin B9 deficiency in obese children and adult populations (34, 35). Although food fortification with folic acid has been increased in most countries, levels of folic acid supplementation are still insufficient for obese individuals. Pravenec M et al. demonstrated that spontaneously hypertensive rats without folic acid intake exhibited a variety of pathological features of MetS, including hepatic ectopic fat accumulation, glucose tolerance, elevated systolic blood pressure, and significant oxidative stress (36). A study by Koo et al. based on the nutritional health survey data from 1,730 pre-menopausal women in Korea found that serum folate levels were significantly associated with MetS prevalence, abdominal obesity, triglyceride levels, and low high-density lipoprotein (37). Another cross-sectional study showed similar results in a group of older adults at higher cardiometabolic risk, with higher folic acid intake associated with lower MetS scores and other metabolic risk factors (38). There are several mechanisms that may have caused this change. Folate deficiency may lead to impaired hepatic methylation, which affects lipid metabolism in the liver (39), and may also cause epigenetic changes with potential implications for the long-term metabolic health of offspring (18, 40).

### 4.3 Vitamin B12 and MetS

Different conclusions were made based on the previous epidemiological studies on the relationship between vitamin B12 and MetS (41, 42). Similar to that of vitamin B9, a linear dose-response relationship was found between the circulating level of vitamin B12 and MetS. Vitamin B12 is absorbed through the intestine and stored in the liver for up to a year. Therefore, the body needs very little vitamin B12, and only strict vegans or those with abnormal metabolism would suffer from a deficiency (43, 44). Although there have been animal or human trials demonstrating the association of vitamin B12 with obesity, dyslipidemia, and elevated pro-inflammatory markers (41, 45, 46), there is a clear lack of studies that can directly demonstrate the correlation between circulating levels of vitamin B12 and MetS risk.

### 4.4 Multivitamin co-exposure and MetS

Due to complicated life scenarios and dietary intake, there is more interest in studying the effects of multivitamin co-exposure on disease. Several studies have found that highly related or similarly acting vitamins have deeper interactions in processes involved in metabolism or disease (47, 48). In our study, we explored the effects of VC, B9, and B12 co-exposure on MetS. The exposure to three water-soluble vitamins was significantly associated with MetS, with each quartile increase in the co-exposure level of water-soluble vitamin concentration associated with a 19% reduction in MetS risk in the conditional model and 16% in the marginal structural model. More importantly, the contribution of each vitamin in reducing MetS risk was varied. In addition, the levels of water-soluble vitamin co-exposure were significantly associated with a decrease in HDL.

Studies have found that high levels of dietary antioxidant capacity, such as a vegan diet, can reduce the risk of MetS. However, a vegan diet can lead to deficiencies in some nutrients, particularly vitamin B12 (49). There is an interaction between vitamin B9 and B12, which has a significant positive effect on improving metabolic syndrome (50). Setola et al. showed that in MetS patients, increased intake of folic acid and vitamin B12 significantly improved insulin resistance and endothelial dysfunction while reducing homocysteine levels (51). In another trial on older patients with hyperhomocysteinemia, these two water-soluble vitamins did not have a positive effect on endothelial function and low inflammatory response in older adults with cardiovascular risk (52). These findings suggest that an appropriate serum vitamin level performs its specific physiological functions while interacting with other vitamins. Our study showed that increased folic acid had a very mild negative effect on reducing the risk of metabolic syndrome when exposed to all three water-soluble vitamins. A similar conclusion was made in a study by Li et al. In obese patients, especially those deficient in vitamin B12, folic acid fortification confers no additional benefit on the patient’s overall health (13).

### 4.5 Strengths and limitations

To the best of our knowledge, this is the first nationally representative study of the association between co-exposure to water-soluble vitamins and MetS. In addition, we performed a number of covariate adjustments, including demographic characteristics and lifestyle factors. However, the study has a few limitations. First, we were unable to draw temporal conclusions between water-soluble vitamin exposure and MetS due to the cross-sectional study design. Second, the vitamin serum concentration test results might be affected by temporary dietary and nutritional supplements because VC, B9, and B12 were absorbed, stored, and metabolized in different ways. Therefore, more rigorously designed trials are needed to further validate conclusions, such as cohort studies or animal experiments to improve quality control and reduce confounding factors.

## 5 Conclusion

This cross-sectional study is the first to examine the association between co-exposure to three important water-soluble vitamins and MetS risk in U.S. adults. The findings suggest that high serum levels of water-soluble vitamins are associated with a reduced risk of MetS. Of the three water-soluble vitamins studied, VC plays an important role in the prevention of MetS. Our findings provide a new way to explore the effects of multiple water-soluble vitamin co-exposure on MetS. Future studies, especially on the potential mechanisms of B9 and B12 interactions with MetS and the specific effects of water-soluble vitamins on different pathological features of MetS are necessary.

## Data availability statement

The datasets presented in this study can be found in online repositories. The names of the repository/repositories and accession number(s) can be found below: https://www.cdc.gov/nchs/nhanes/index.htm.

## Ethics statement

The studies involving human participants were reviewed and approved by the Research Ethics Review Board of the American National Center for Health Statistics. The patients/participants provided their written informed consent to participate in this study.

## Author contributions

HS and AT: conception and design of the study. XP, JY and SR: acquisition and interpretation of data, drafting the article. HL, SY and YZ: formal analysis and Methodology. MW: draw figures and tables, drafting the article. All authors contributed to and have approved the submitted version.

## Supplementary material

Supplementary Figure 1 Quantile g-computation model regression index weighs **(A)** and joint effect **(B)** (95% confidence interval) of fat-soluble vitamins (i.e., Vitamin C, Vitamin B9, and Vitamin B12) on waist circumference. Waist circumference was categorized into low (<102 cm in men or <88 cm in women) and high (≥102 cm in men or ≥88 cm in women) groups, and the low group was used as the reference.

Supplementary Figure 2 Quantile g-computation model regression index weighs **(A)** and joint effect **(B)** (95% confidence interval) of fat-soluble vitamins (i.e., Vitamin C, Vitamin B9, and Vitamin B12) on triglyceride. Triglyceride was categorized into low (<150 mg/dL) and high (≥150 mg/dL) groups, and the low group was used as the reference

Supplementary Figure 3 Quantile g-computation model regression index weighs **(A)** and joint effect **(B)** (95% confidence interval) of fat-soluble vitamins (i.e., Vitamin C, Vitamin B9, and Vitamin B12) on high-density lipoprotein. High-density lipoprotein was categorized into low (<40 mg/dL in men or <50 mg/dL in women) and high (≥40 mg/dL in men or ≥50 mg/dL in women) groups, and the high group was used as the reference

Supplementary Figure 4 Quantile g-computation model regression index weighs **(A)** and joint effect **(B)** (95% confidence interval) of fat-soluble vitamins (i.e., Vitamin C, Vitamin B9, and Vitamin B12) on blood pressure. Blood pressure was categorized into low (systolic blood pressure <130 mmHg and diastolic blood pressure<85 mmHg) and high (systolic blood pressure ≥130 mmHg or diastolic blood pressure ≥85 mmHg) groups, and the low group was used as the reference;

Supplementary Figure 5 Quantile g-computation model regression index weighs **(A)** and joint effect **(B)** (95% confidence interval) of fat-soluble vitamins (i.e., Vitamin C, Vitamin B9, and Vitamin B12) on fasting plasma glucose. Fasting plasma glucose was categorized into low (<110 mg/dL) and high (≥110 mg/dL) groups, and the low group was used as the reference.
