# Supplementary figures and images for: Association of serum water-soluble vitamin exposures with the risk of metabolic syndrome: results from NHANES 2003-2006
Source: Front Endocrinol (Lausanne). 2023 May 12;14:1167317. doi: 10.3389/fendo.2023.1167317 (PMC10213561; doi:10.3389/fendo.2023.1167317)

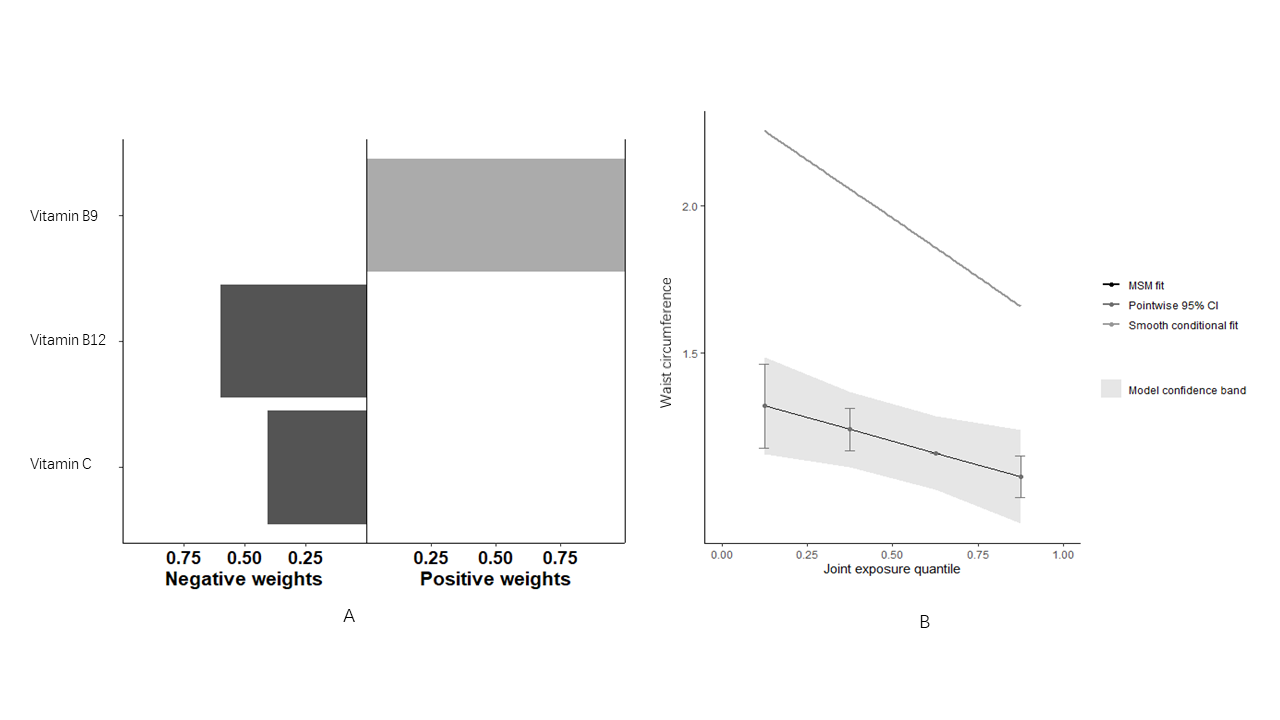

Supplement: Supplementary Figure 1 — Quantile g-computation model regression index weighs (A) and joint effect (B) (95% confidence interval) of fat-soluble vitamins (i.e., Vitamin C, Vitamin B9, and Vitamin B12) on waist circumference. Waist circumference was categorized into low (<102 cm in men or <88 cm in women) and high (≥102 cm in men or ≥88 cm in women) groups, and the low group was used as the reference. [file Image_1.tif]

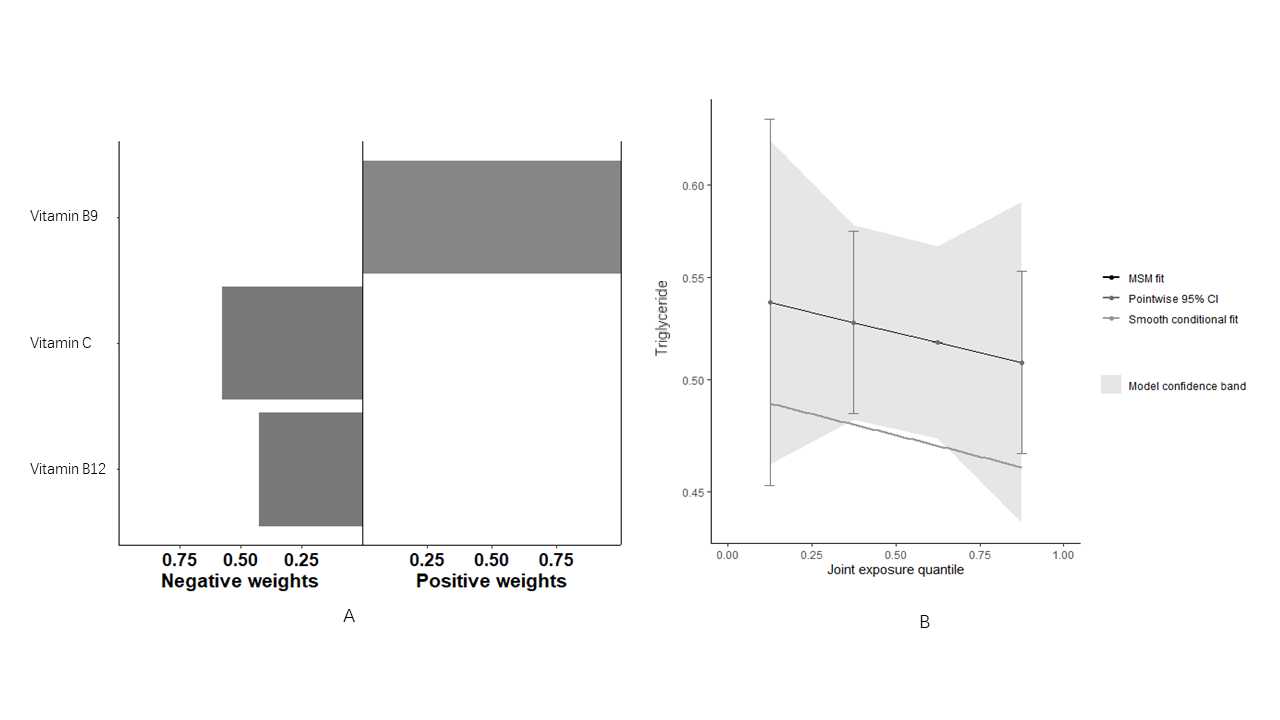

Supplement: Supplementary Figure 2 — Quantile g-computation model regression index weighs (A) and joint effect (B) (95% confidence interval) of fat-soluble vitamins (i.e., Vitamin C, Vitamin B9, and Vitamin B12) on triglyceride. Triglyceride was categorized into low (<150 mg/dL) and high (≥150 mg/dL) groups, and the low group was used as the reference [file Image_2.tif]

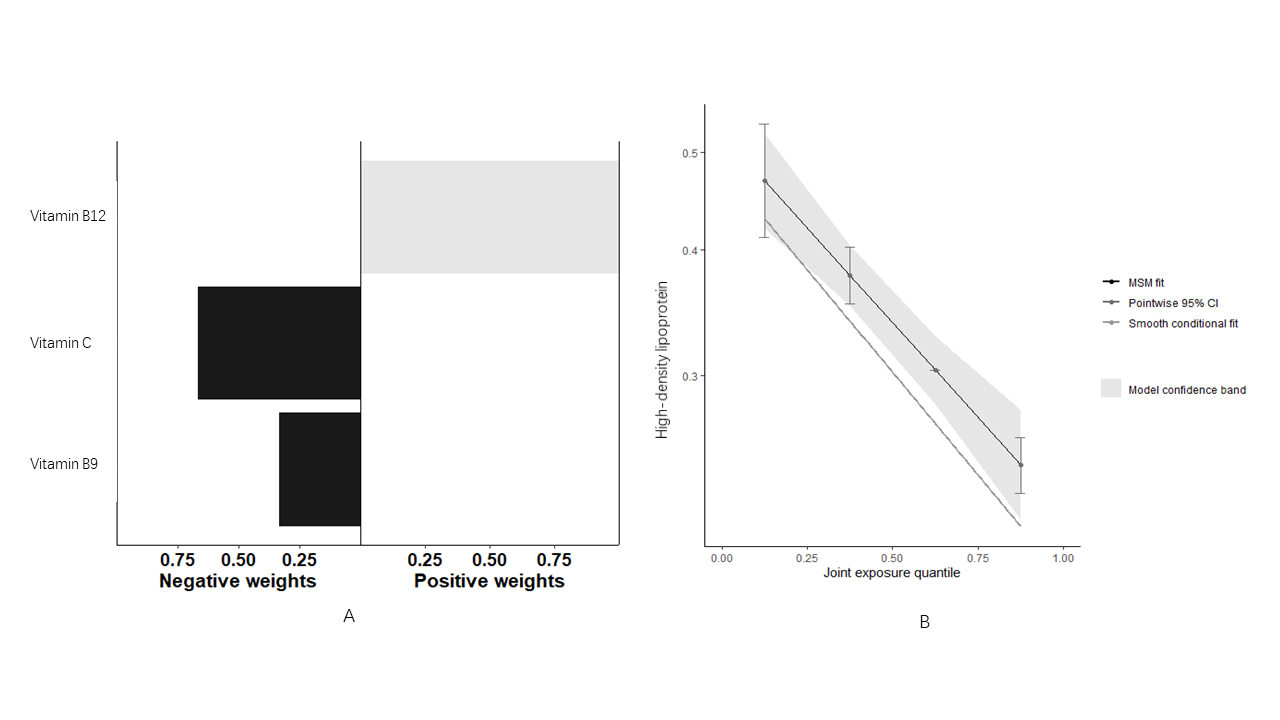

Supplement: Supplementary Figure 3 — Quantile g-computation model regression index weighs (A) and joint effect (B) (95% confidence interval) of fat-soluble vitamins (i.e., Vitamin C, Vitamin B9, and Vitamin B12) on high-density lipoprotein. High-density lipoprotein was categorized into low (<40 mg/dL in men or <50 mg/dL in women) and high (≥40 mg/dL in men or ≥50 mg/dL in women) groups, and the high group was used as the reference [file Image_3.tif]

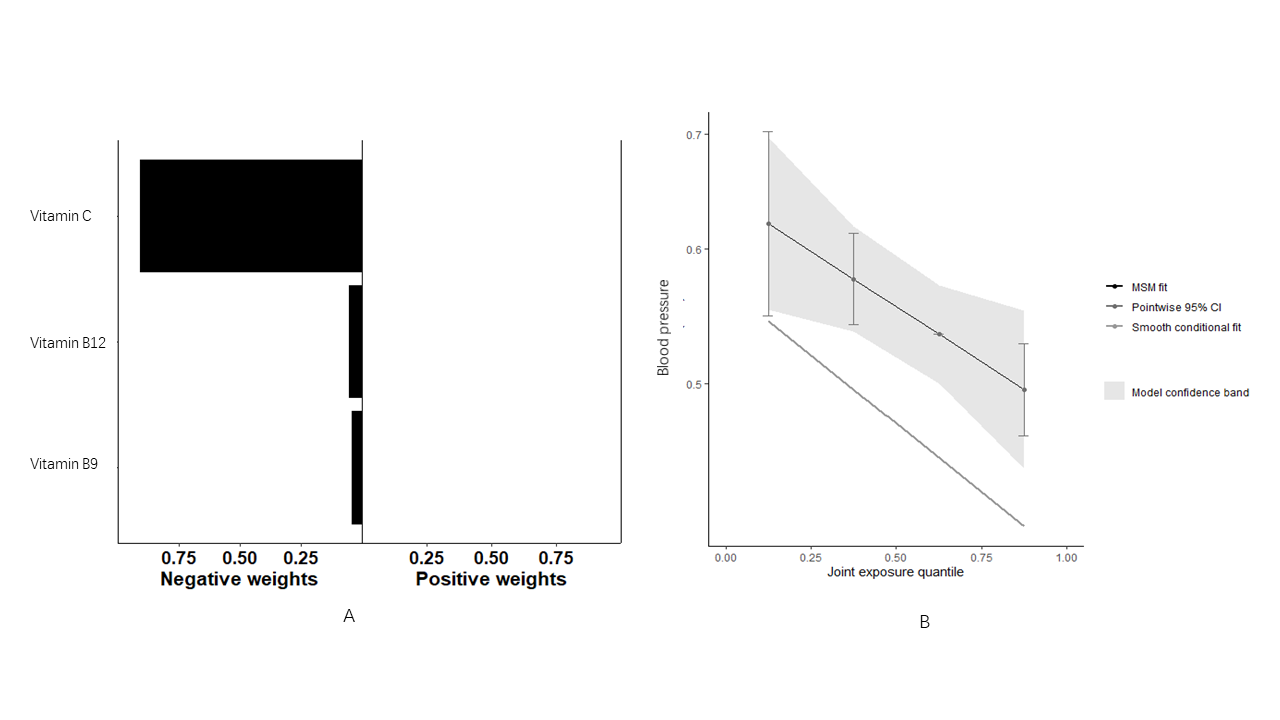

Supplement: Supplementary Figure 4 — Quantile g-computation model regression index weighs (A) and joint effect (B) (95% confidence interval) of fat-soluble vitamins (i.e., Vitamin C, Vitamin B9, and Vitamin B12) on blood pressure. Blood pressure was categorized into low (systolic blood pressure <130 mmHg and diastolic blood pressure<85 mmHg) and high (systolic blood pressure ≥130 mmHg or diastolic blood pressure ≥85 mmHg) groups, and the low group was used as the reference; [file Image_4.tif]

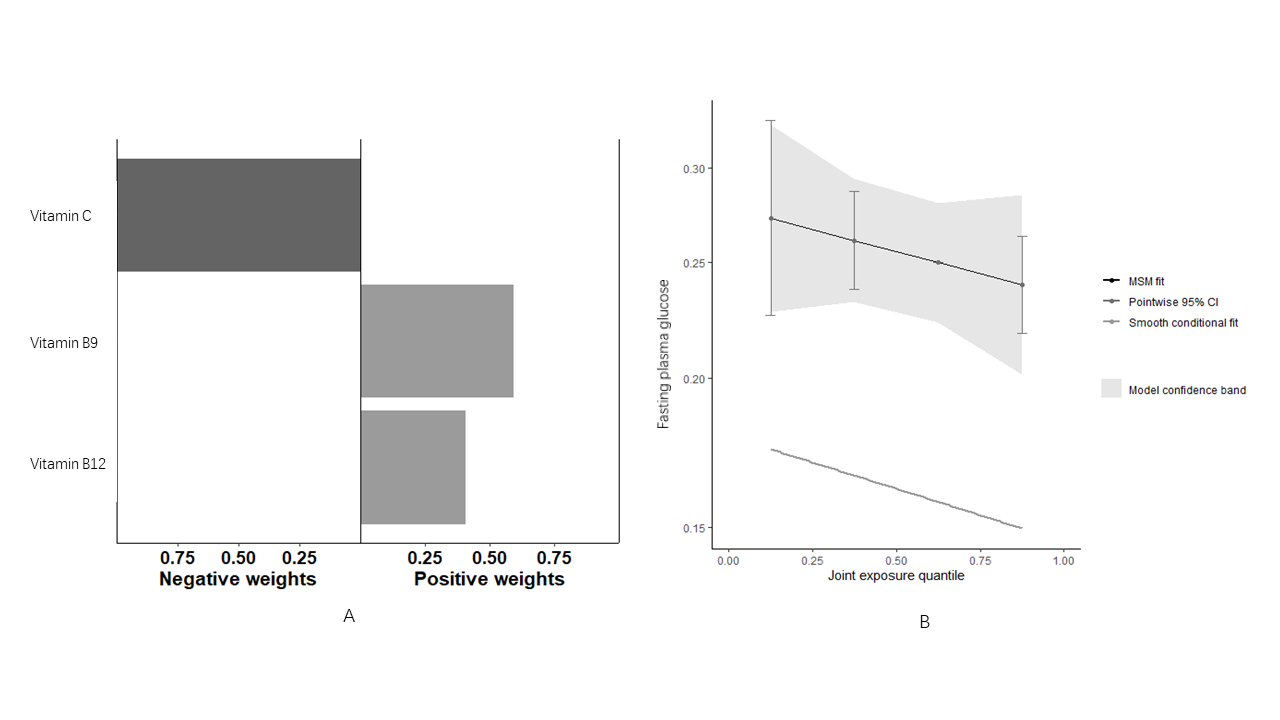

Supplement: Supplementary Figure 5 — Quantile g-computation model regression index weighs (A) and joint effect (B) (95% confidence interval) of fat-soluble vitamins (i.e., Vitamin C, Vitamin B9, and Vitamin B12) on fasting plasma glucose. Fasting plasma glucose was categorized into low (<110 mg/dL) and high (≥110 mg/dL) groups, and the low group was used as the reference. [file Image_5.tif]
