# Supplementary material for: Association of serum water-soluble vitamin exposures with the risk of metabolic syndrome: results from NHANES 2003-2006
Source: Front Endocrinol (Lausanne). 2023 May 12;14:1167317. doi: 10.3389/fendo.2023.1167317 (PMC10213561; doi:10.3389/fendo.2023.1167317)
Supplement: Supplementary file 6 [file Table_1.docx]

Table S1. Associations of individual serum fat-soluble vitamins exposure with each component of metabolic syndromes in NHANES 2003-2006 participants.

| Vitamin | Quantile 1 | Quantile 2 | Quantile 3 | Quantile 4 | *P*_continuous_ |
| --- | --- | --- | --- | --- | --- |
| *Waist circumference* | | | | | |
| Model1 |  |  |  |  |  |
| Vitamin C | 1 | 0.90 (0.60, 1.36) | 0.65 (0.46, 0.94) | 0.67 (0.44, 1.04) | 0.0456 |
| Vitamin B9 | 1 | 1.02 (0.73, 1.42) | 1.23 (0.90, 1.67) | 1.15 (0.77, 1.71) | 0.1058 |
| Vitamin B12 | 1 | 0.96 (0.68, 1.36) | 1.06 (0.75, 1.49) | 0.99 (0.65, 1.49) | 0.8264 |
| Model2 |  |  |  |  |  |
| Vitamin C | 1 | 0.87 (0.57, 1.34) | 0.63 (0.43, 0.93) | 0.70 (0.45, 1.09) | 0.0673 |
| Vitamin B9 | 1 | 1.04 (0.74, 1.45) | 1.16 (0.84, 1.60) | 1.11 (0.75, 1.64) | 0.1665 |
| Vitamin B12 | 1 | 0.89 (0.63, 1.26) | 1.07 (0.76, 1.49) | 0.93 (0.61, 1.43) | 0.8344 |
| *Triglyceride* | | | | | |
| Model1 |  |  |  |  |  |
| Vitamin C | 1 | 0.76 (0.58, 1.01) | 0.96 (0.72, 1.28) | 0.70 (0.53, 0.91) | 0.0466 |
| Vitamin B9 | 1 | 1.08 (0.83, 1.39) | 1.18 (0.91, 1.53) | 1.11 (0.84, 1.45) | 0.8640 |
| Vitamin B12 | 1 | 1.00 (0.77, 1.29) | 1.00 (0.78, 1.28) | 0.82 (0.63, 1.05) | 0.1253 |
| Model2 |  |  |  |  |  |
| Vitamin C | 1 | 0.82 (0.62, 1.10) | 1.04 (0.77, 1.40) | 0.73 (0.55, 0.96) | 0.1680 |
| Vitamin B9 | 1 | 1.09 (0.84, 1.40) | 1.20 (0.90, 1.60) | 1.12 (0.87, 1.45) | 0.9480 |
| Vitamin B12 | 1 | 0.99 (0.77, 1.28) | 1.01 (0.78, 1.31) | 0.81 (0.63, 1.04) | 0.1551 |
| *High-density lipoprotein* | | | | | |
| Model1 |  |  |  |  |  |
| Vitamin C | 1 | 0.87 (0.67, 1.14) | 0.71 (0.54, 0.94) | 0.45 (0.30, 0.65) | <0.0001 |
| Vitamin B9 | 1 | 0.86 (0.66, 1.11) | 0.80 (0.60, 1.05) | 0.66 (0.46, 0.94) | 0.0179 |
| Vitamin B12 | 1 | 1.21 (0.90, 1.62) | 0.86 (0.64, 1.15) | 0.93 (0.73, 1.19) | 0.1054 |
| Model2 |  |  |  |  |  |
| Vitamin C | 1 | 0.93 (0.73, 1.20) | 0.76 (0.57, 1.01) | 0.47 (0.32, 0.71) | 0.0004 |
| Vitamin B9 | 1 | 0.88 (0.68, 1.14) | 0.79 (0.60, 1.04) | 0.68 (0.47, 0.97) | 0.0259 |
| Vitamin B12 | 1 | 1.22 (0.90, 1.65) | 0.89 (0.66, 1.20) | 0.95 (0.73, 1.24) | 0.1952 |
| *Blood pressure* | | | | | |
| Model1 |  |  |  |  |  |
| Vitamin C | 1 | 0.76 (0.60, 0.97) | 0.67 (0.52, 0.87) | 0.65 (0.48, 0.87) | 0.0004 |
| Vitamin B9 | 1 | 0.87 (0.65, 1.16) | 0.82 (0.63, 1.07) | 0.92 (0.67, 1.26) | 0.5661 |
| Vitamin B12 | 1 | 0.99 (0.69, 1.43) | 0.91 (0.71, 1.18) | 0.90 (0.66, 1.23) | 0.0539 |
| Model2 |  |  |  |  |  |
| Vitamin C | 1 | 0.71 (0.54, 0.93) | 0.63 (0.49, 0.80) | 0.60 (0.43, 0.83) | 0.0005 |
| Vitamin B9 | 1 | 0.85 (0.63, 1.16) | 0.79 (0.61, 1.03) | 0.88 (0.64, 1.22) | 0.3974 |
| Vitamin B12 | 1 | 0.98 (0.68, 1.42) | 0.89 (0.70, 1.14) | 0.88 (0.65, 1.19) | 0.0331 |
| *Fasting plasma glucose* | | | | | |
| Model1 |  |  |  |  |  |
| Vitamin C | 1 | 0.97 (0.74, 1.26) | 0.68 (0.53, 0.89) | 0.79 (0.57, 1.10) | 0.1452 |
| Vitamin B9 | 1 | 0.98 (0.69, 1.41) | 1.08 (0.74, 1.57) | 1.19 (0.86, 1.64) | 0.3719 |
| Vitamin B12 | 1 | 1.27 (0.86, 1.88) | 0.92 (0.62, 1.35) | 1.25 (0.82, 1.89) | 0.489 |
| Model2 |  |  |  |  |  |
| Vitamin C | 1 | 1.03 (0.77, 1.38) | 0.70 (0.52, 0.94) | 0.81 (0.57, 1.16) | 0.2973 |
| Vitamin B9 | 1 | 1.03 (0.71, 1.48) | 1.04 (0.70, 1.53) | 1.18 (0.86, 1.62) | 0.4081 |
| Vitamin B12 | 1 | 1.30 (0.87, 1.94) | 0.92 (0.63, 1.35) | 1.23 (0.82, 1.86) | 0.4722 |

NHANES, the National Health and Nutrition Examination Survey.

All estimates were calculated by multivariable logistic regression model, and results were expressed as odds ratio (95% confidence interval). Covariates adjusted in model 1 included age, sex, ethnicity, education, marital status, body mass index and ratio of family income to poverty. Covariates adjusted in model 2 included leisure time physical activity, dietary energy, drinking status and smoking status in addition to those in model 1.

*P*_continuous_ meant the *P* value of multivariable logistic regression model using continuous Vitamins levels as exposure.

Each component of metabolism syndromes was categorized based on NCEP: ATP III guidelines. Waist circumference was categorized into low (<102 cm in men or <88 cm in women) and high (≥102 cm in men or ≥88 cm in women) groups, and low group was used as reference; triglyceride was categorized into low (<150 mg/dL) and high (≥150 mg/dL) groups, and low group was used as reference; high-density lipoprotein was categorized into low (<40 mg/dL in men or <50 mg/dL in women) and high (≥40 mg/dL in men or ≥50 mg/dL in women) groups, and high group was used as reference; blood pressure was categorized into low (systolic blood pressure <130 mmHg and diastolic blood pressure<85 mmHg) and high (systolic blood pressure ≥130 mmHg or diastolic blood pressure ≥85 mmHg) groups, and low group was used as reference; fasting plasma glucose was categorized into low (<110 mg/dL) and high (≥110 mg/dL) groups, and low group was used as reference.
